# Novel Octa‐Structure Metamaterial Architecture for High Q‐Factor and High Sensitivity in THz Impedance Spectroscopy

**DOI:** 10.1002/advs.202407824

**Published:** 2024-10-30

**Authors:** Heena Khand, Rudrarup Sengupta, Gabby Sarusi

**Affiliations:** ^1^ Department of Photonics and Electro‐Optics Engineering School of Electrical and Computer Engineering Ben‐Gurion University of the Negev Beer Sheva 84105 Israel

**Keywords:** dielectric response, Metamaterial resonance, Octa‐Structure Architecture, sensitivity enhancement, THz impedance spectroscopy

## Abstract

Terahertz (THz) electric inductive‐capacitive (ELC) resonant metamaterials (MMs) are well established tools that can be used to detect the presence of dielectric material (e.g., nanoparticles, bioparticles, etc.) spread on their surfaces within the gap of the capacitive plates of a nanoantenna array. In THz spectroscopy, the amount of the red shift in the resonance frequency (Δ*F*) plays an important role in the detection of nanoparticles and their concentration. We introduce a new LC resonant MM architecture in the ELC category that maximizes dielectric sensitivity. The newly proposed architecture has an octahedral structure with uniform capacitive gaps at each forty‐five‐degree interval, making the structure super‐symmetric and polarization independent. The inductor core is condensed into a central solid circle connecting all the eight lobes of the octahedron, thereby completing the LC circuit. This ELC resonator has very large active areas (capacitor‐gaps), with hotspots at the periphery of each unit cell. The MM structure is repeated in a clustered fashion, so that the peripheral hotspots are also utilized in dielectric sensing. This results in enhancing the quality factor of MM resonance, as well as in increasing Δ*F*. The research comprises a combination of rigorous system‐level simulations along with THz impedance spectroscopy laboratory experiments. We achieved a highly sensitive MM sensor with sensitivity reaching 1600 GHz/RIU. This sensor is fully CMOS compatible and has promising potential applications in high‐sensitivity bio‐sensing, characterization of nanoparticles, and ultra‐low‐concentration dielectrics detection, as well as in sensing differential changes in the composition of substances deposited on the metasurface.

## Graphical abstract

A new highly sensitive ELC THz metamaterial resonator with a super‐symmetric octa‐structure architecture is developed for ultrasensitive dielectric sensing. The architecture has much lower inductive elements and utilizes the peripheral high electric field regions as capacitor‐gaps and true hotspots for dielectric sensing. This architecture reaches sensitivity of 1600GHz/RIU, which is one of the highest for a dielectric sensing resonator till‐date.

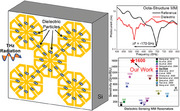

## 1 Introduction

Over the last two decades, there have been profound advances in the field of terahertz (THz) science and technology. Numerous developments in the fields ofTHz sources and detectors,^[^ ^1^, ^2^, ^3^, ^4^, ^5^ ^]^ THz metamaterial (MM) sensors,^[^ ^6^, ^7^, ^8^, ^9^, ^10^, ^11^, ^12^, ^13^ ^]^ THz filtering, modulation and switching devices,^[^ ^14^ ^]^ THz imaging,^[^ ^2^, ^15^ ^]^ THz material characterization (biological, chemical, medical, pharmaceutical),^[^ ^16^, ^17^, ^18^, ^19^, ^20^, ^21^, ^22^, ^23^, ^24^, ^25^ ^]^ THz security inspection^[^ ^26^, ^27^ ^]^ and THz spectroscopy for the detection of diseases such as cance^[^ ^28^, ^29^, ^30^, ^31^ ^]^ and lung‐related diseases^[^ ^32^, ^33^ ^]^ have been reported. THz MMs of appropriate size and architecture generate resonances that interact with the THz radiation incident on the periodic metasurface, causing the plasmons to oscillate uniformly at the resonant frequency.^[^ ^22^ ^]^ Therefore, introducing any material on a MM surface may further increase the capacitance, and hence red‐shift the resonance frequency (Δ*F*  − spectral shift), which can be detected utilizing impedance spectroscopy.^[^ ^32^, ^33^, ^34^ ^]^ For instance, the linear single split ring resonator (SRR) is a popular electric inductive‐capacitive (ELC) circuit‐based MM structure used for the detection of nano‐to‐micro sized particles.^[^ ^35^, ^36^, ^37^ ^]^

The resonance strength of the MM is an important characteristic determining the efficiency, resonance quality factor and absorption/reflection magnitude at the resonance frequency.^[^ ^34^, ^38^, ^39^ ^]^ Several techniques for improving the resonance strength of MMs by tailoring structural parameters such as the architecture of the MM,^[^ ^32^, ^40^ ^]^ optical property variations (of MM metals, dielectrics and substrates),^[^ ^41^, ^42^ ^]^ the thickness of the MM substrate,^[^ ^43^, ^44^ ^]^ dark mode resonances and Fano resonances^[^ ^45^, ^46^, ^47^, ^48^ ^]^ can be found in the literature. The dielectric particle sensitivity of the MM (dielectric response) is represented by the proportional factor, namely, Δ*F* of the resonance frequency when nano‐to‐micro sized particles are inserted in the capacitive‐gap (cap‐gap) of the MM.^[^ ^32^, ^35^ ^]^ Various published researches discuss applications of MMs as sensors that detect dielectrics of various sizes and concentrations.^[^ ^32^, ^33^, ^34^, ^35^, ^36^, ^37^, ^49^ ^]^ The sensitivity, expressed as Δ*F*, is primarily determined by the electromagnetic field strength on the MM surface at resonance.^[^ ^34^, ^38^ ^]^ Two further important parameters of the THz MM are the spectral stability of resonance and its polarization invariance. In THz spectroscopy analysis, opting for polarization‐independent MMs is of great importance.^[^ ^32^, ^33^, ^34^ ^]^ Our goal in this research is to investigate a novel design of THz MMs that meets all of the above requirements, namely, achieves extremely sensitive dielectric responses by maximizing the electrical field on the MM surface, by making the MM architecture super‐symmetrical to avoid polarization dependency, and by maximizing the active areas (cap‐gaps) (which is especially useful for low‐density particle sensing).

In our previous works, we introduced a polarization‐independent four‐arrowhead LC resonant MM with cap‐gaps along the diagonals of a square SRR MM structure, which displayed an improved active area compared to those of the linear LC resonant SRRs.^[^ ^12^, ^35^, ^37^ ^]^ We achieved increased MM sensitivity by increasing the probability of nanoparticles falling within the active region of the cap‐gap.^[^ ^40^ ^]^ We also demonstrated a prompt SARS‐CoV‐2 screening test that is based on the detection of Δ*F* caused by related exhaled particles, which yielded 88% agreement with the RT‐qPCR results for the alpha to delta variants of SARS‐CoV‐2.^[^ ^32^ ^]^ Further, we reported on the existence of an electromagnetic coupling mechanism between the substrate's Fabry‐Pérot (FP) oscillations and the MM resonance, offering a new paradigm to physically increase the MM sensitivity upon their decoupling.^[^ ^34^ ^]^ We showed that unwanted coupling in standard Si substrates can be eliminated after a thinning process (CMOS compatible), giving rise to a large MM resonance shift and thus achieving higher sensitivity. We developed two sensitivity‐enhancement techniques, one involving backside thinning^[^ ^34^ ^]^ and another involving optically attaching dielectric back‐plates (BPs) to the backside of the substrate.^[^ ^50^ ^]^ Thinning of the substrate broadens the FP periodicity and the BPs reduce the absorption in free spectral range (FSR) regions of the FP.^[^ ^50^ ^]^ The combined effect of thinning and BP attachment completely removes the substrate losses and maximizes the dielectric sensitivity.^[^ ^50^ ^]^ Yet, there remains room for further sensitivity enhancement that is associated with the basic architecture of the MM building block.

In this work, we introduce a new LC resonant MM architecture in the ELC category that aims to further improve the dielectric sensing performance. Unlike the previous four‐arrowhead architecture, the proposed architecture has an octahedral structure with a larger area of cap‐gaps at each forty‐five‐degree interval, making the structure super‐symmetric and polarization independent. The inductor core is condensed into a circular central unit that connects all the eight lobes of the octahedron, thereby completing the LC circuit. This ELC resonator has a very large active area (cap‐gaps), with hotspots at the periphery of each unit cell. The MM structure is repeated in a clustered fashion, so that the peripheral hotspots are also utilized for dielectric sensing. Based on the simulations performed, this new architecture provides a more than two‐fold increase in sensitivity compared to our previous arrowhead MM and is twenty‐six times more sensitive than the linear ELC SRR. By utilizing further sensitivity enhancement techniques of decoupling FP‐MM resonances^[^ ^34^, ^50^ ^]^ in this work, we have achieved very high sensitivities with our super‐symmetric octa‐metastructure.

## 2 Octa‐Structure Architecture

**Figure 1:**
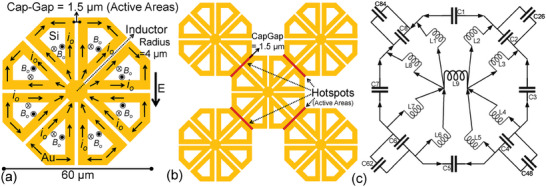
(a) Schematic of the octa‐structure ELC resonator unit cell is shown with dimensions in micrometers. The calculated direction of current (*i*_0_) at *F_reso_* (at a time instant) under the influence of external electric field (*E*) is shown. The nullified magnetic flux density (*B*_0_) in all the lobes is also shown. (b) Schematic of the octa‐structure ELC resonator unit cell arranged in clustering fashion, with surrounding unit cells placed at a distance equal to that of the cap‐gap. Peripheral hotspots used as active areas (cap‐gaps) are marked in red. (c) Equivalent circuit diagram of the octa‐structure MM is shown with all the inductors numbered from *L*_1_ to *L*_8_ (clockwise) and capacitors numbered from *C*_1_ to *C*_8_ (clockwise), with peripheral capacitors marked as *C*_26_, *C*_48_, *C*_62_, and *C*_84_ with the arrows indicating current flow.

Our novel octahedral planar ELC THz resonator architecture was inspired by the microwave absorber eight circular sector structure.^[^ ^51^, ^52^ ^]^ For an absorber, when the MM impedance and the free space impedance become equal, the reflection coefficient becomes zero.^[^ ^51^, ^52^ ^]^ This leads to antiparallel currents between the top and bottom of the MM unit cell, creating a magnetic dipole.^[^ ^51^, ^52^ ^]^ In contrast, for an ELC resonator, an opposite magnetic dipole is generated on the surface of the resonator, resulting in zero magnetoelectric coupling.^[^ ^53^, ^54^ ^]^ We developed the THz ELC resonator by engineering the MM in a way such that for an electric excitation, opposite magnetic dipoles are generated in each lobe of the octa‐structure, resulting in a net zero magnetic dipole (shown in **Figure** **1**a), and evoking a pure electric response. Supplementary information section 1 shows the details of the step‐by‐step architectural engineering development of our ELC resonator.

Figure 1a shows a schematic of our octa‐structure ELC resonator. Each unit cell has a diameter of 60 µm. The eight lobes of the unit cell are separated by uniform cap‐gaps of 1.5 µm, which has been shown to be the most suitable gap for sub‐micron and micron size particle sensing in THz spectroscopy.^[^ ^32^, ^33^ ^]^ With a cap‐gap of 1.5 µm, the MM will have the capability to sensitively detect viruses and different nanoparticles (e.g., ZnO, PbN, etc.), proteins, and a variety of other particles of sizes below 1.5 µm. There are cap‐gaps for nanoparticles and dielectric sensing at each forty‐five‐degree interval, which greatly increases the probability of particle sensing when dealing with low‐density dielectrics. Each of these lobes is connected to a central core inductor of 4µm in radius, which helps to complete the resonant circuit, as can be seen in Figure 1C. The resonant frequency (*F_reso_*) can be calculated using the LC resonance antenna equation $F_{reso}=1/2\pi \sqrt{LC}$, where *L* is the effective inductance and *C* is the effective capacitance. This will fall within the THz regime with the sizes listed in Figure 1a. Increasing the diameter of the unit cell increases the effective inductance (*L*) and capacitance (*C*), thus decreasing its resonance frequency; however, increasing the inner radius of the inductor core decreases the effective capacitance (*C*) and active area, which, in turn, increases *F_reso_*. On the other hand, increasing the cap‐gap width decreases the effective capacitance (*C*), which, in turn, increases *F_reso_*. The final geometry of the resonator is as per Figure 1a, and resonates at a frequency of 700 GHz when fabricated on a Si substrate and a 100 nm thick gold (Au) layer. All THz MM spectral simulations were done using the finite‐element CST Studio Suite simulator in the frequency domain mode.

Figure 1a also shows the simulated current directions at the resonance frequency (at a time instant) under the influence of an external electric field. The induced current *i*_0_ from the THz spectrometer radiation flows through each lobe of the octahedron and passes through the central inductor core. The central inductor plays an important role, acting like a nucleus such that all the currents pass through it. The external electric field oscillations from the spectrometer generate clockwise and anticlockwise currents in each lobe, which nullifies the magnetic flux density (*B*_0_) in all the lobes and in the entire structure, as depicted in figure 1a. If we compare our octa‐structure to the absorber eight circular sector structure, we can observe that the metal is deposited only on the perimeter of each lobe of the octahedron. This novel design helps to generate the clockwise and anticlockwise currents in each lobe, thereby nullifying the net magnetoelectric coupling and making the structure ELC resonant (details are given in supplementary information section 1).

Like any other resonating SRR, the external electric field oscillations induce dipole‐formation in the eight active areas. Studying the currents approaching the periphery of the octahedron, it is evident that they originated and terminated at the peripheral edges, as depicted in Figure 1a. Therefore, each of the peripheral edges will have an electric field hotspot, which will generate high density electric diploes. Therefore, we place four adjacent octahedron unit cells in close proximity, generating a large area of octa‐structure building blocks so that we can utilize the peripheral electric field hotspot as an active area with the same cap‐gap. The octa‐structure MM duplicated on the entire surface is presented in Figure 1b. We deliberately placed the surrounding octahedral unit cells at the same distance as the cap‐gap (1.5 µm). Unlike a conventional hexagonal honeycomb, in this case it is geometrically possible to place only four unit‐cells adjacent to each other in a clustered octa‐structure, thereby utilizing the full potential of the metasurface area. Therefore, all the eight cap‐gaps in Figure 1a, in addition to the four peripheral hotspots shown in Figure 1b, constitute the total active areas. Interestingly, other published MM structures with pentagonal or hexagonal etc. shapes^[^ ^52^ ^]^ have more metal on the surface and less capacitive area compared to our octa‐structure; more importantly, the structures are not super‐symmetric, and thus are polarization dependent. Our proposed octa‐structure, along with the peripheral cap‐gap, will be completely polarization independent (this will be proved later in the experimental section), which is a major advantage when using polarized THz source spectrometers.

Figure 1c shows the equivalent circuit diagram of the octa‐structure MM. Each octa‐lobe is one inductor numbered from *L*_1_ to *L*_8_ (clockwise). All the inductors connect to the central inductor core *L*_9_. Similarly, each of the eight capacitors are numbered from *C*_1_ to *C*_8_ (clockwise). The peripheral capacitors are connected in parallel to the capacitors *C*_2_, *C*_4_, *C*_6_, and *C*_8_, and are numbered *C*_26_, *C*_48_, *C*_62_, and *C*_84_, respectively. The external magnetic oscillation due to the incident electromagnetic field induces identical looping currents in each lobe, resulting in net zero magnetic flux density in the metastructure at the resonant frequency.

We have developed interacting octahedral unit cells, placed in a clustered octahedral fashion for maximum utilization of the active metasurface area, for the purpose of highly sensitive dielectric particle sensing. Compared to the arrowhead structure or the well‐known linear ELC SRR,^[^ ^35^ ^]^ we have increased the active area substantially, and minimized the inductor surface area.

## 3 Simulations

**Figure 2:**
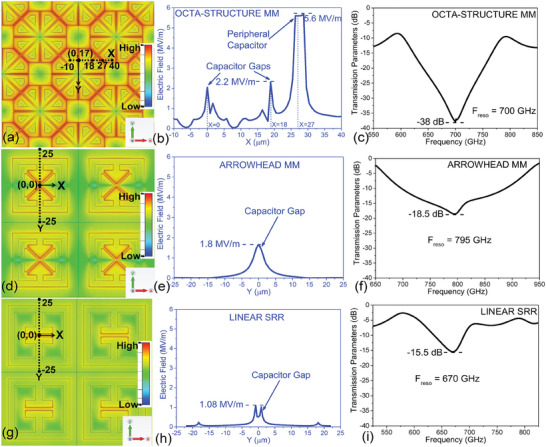
CST simulation of the electric field density plotted on the metasurface at the resonance frequency (F_reso_), for (a) octa‐structure MM, *F_reso_* =  700 *GHz*, (d) arrowhead MM, *F_reso_* =  795 *GHz*, and (g) linear SRR, *F_reso_* =  670 *GHz*. For each of the MMs, a cross section taken on the metasurface is shown by black dotted lines from which the electric field is extracted. (b) Extracted electric field for octa‐structure MM is plotted along the X axis at *Y* = 17 µ*m*, showing the field intensity at the cap‐gaps (*x* = 0 µ*m* and *x* = 18 µ*m*) and at the peripheral cap‐gap (*x* = 27 µ*m*). Extracted electric fields for (e) arrowhead MM and (h) linear SRR are plotted along the Y axis at *x* = 0 µ*m*, showing the field intensity at the cap‐gap (*y* = 0 µ*m*). Transmission parameters (in dB) are plotted for (c) octa‐structure MM, (f) arrowhead MM, and (i) linear SRR, including F_reso_ and resonance depths in dB. All the plots (a), (d), (g); (b), (e), (h); and (c), (f), (i) are drawn to the same scales (over different respective ranges) for easy comparison.

To simulate the electric fields on the metasurface, we used CST Studio Suite (finite‐element electromagnetic solver). In **Figure** **2**A, we show the 2D electric fields on the metasurface of our octa‐structure MM at resonance frequency *F_reso_* = 700 GHz. We can observe that the electric field density is considerably higher in the active areas, consisting of the internal and peripheral cap‐gaps. To calculate the exact value of the field intensity in the active areas, we took a cross‐section on the metasurface at *y* = 17 µm, and *x* = − 10 *to* 40 µm (in Figure 2a). Accordingly, we observe that we get the internal cap‐gaps at *x* = 0 µm, and at *x* = 18 µm, and the peripheral cap‐gap at *x* = 27 µm. This specific cross section was selected in order to assess the electric field intensity at multiple internal cap‐gaps and at least one peripheral cap‐gap. The electric field for this cross‐section along the X axis at *y* = 17 µm is given in Figure 2b. We observe two peaks of the electric field of 2.2 MV/m at the internal cap‐gap locations (*x*  = 0 µm, and *x* = 18 µm). Interestingly, at the peripheral cap‐gap (*x* = 27 µm) we observe a much larger field intensity peak of 5.6 MV/m, which is more than 2.5 times larger than the field peak of the internal cap‐gaps.

The electric field density at *F_reso_* in the cap‐gap region is directly correlated to the electric dipole strength, which indicates the more intense plasmonic effect. Looking at the current flow pattern in Figure 1a, we observe that the peripheral edge of the octahedral metastructure contains field hotspots; the simulation results help to quantify our claim. We expect intense plasmonic interaction when the octa‐structures are used in the interacting and clustered fashion, because the hotspot areas are used as capacitors. Evidence of high plasmonic interaction of the metasurface with the THz radiation can be seen in Figure 2c, where we have plotted the transmission parameters, showing the resonance frequency of the octa‐structure MM. The figure also shows the transmittance at resonance, which was found to be ‐38 dB. A lower transmittance at resonance for ELC resonators enhances blockage of electromagnetic radiation, which is further manifested in high plasmonic interaction of the metasurface. Higher plasmonic interaction is directly correlated with dielectric sensitivity^[^ ^34^ ^]^ for ELC resonators, and hence we should expect significant resonance frequency red shifts, even for small concentrations of dielectric particles. These results will become more robust when we compare them with other well‐established MM architectures for dielectric sensing.

In order to compare this result of the octa‐structure MM with other MM architectures, we performed similar simulations on a previously‐designed four arrowhead MM,^[^ ^32^ ^]^ and on the classical linear SRR,^[^ ^35^ ^]^ with the same thickness of silicon substrate and metallization of 100 nm Au, and 1.5 µm cap‐gaps. The electric field distribution over the metasurface for the arrowhead MM at *F_reso_* =  795 *GHz* is shown in Figure 2d; we took a cross section at *x* = 0, and plotted the electric field from *y* = − 25 *to*  + 25 µm in Figure 2e. At the cap‐gap (*x* = 0, *y* = 0) we observe a field intensity peak of 1.8 MV/m. This field intensity is 1.2 times lower than that of the internal cap‐gap field and 3.1 times lower than the peripheral cap‐gap field of the octa‐structure MM. Also, the transmittance at resonance, given in Figure 2f, is ‐18.5 dB, which is more than twice (in dB) that for the octa‐structure MM, indicating lower plasmonic interaction. Similarly, the electric field distribution over the metasurface for a linear SRR at *F_reso_* =  670 *GHz* is shown in Figure 2g; we took a cross section at *x* = 0, and plotted the electric field from *y* = − 25 to  + 25 µm. At the cap‐gap region (*x* = 0,  *y* = 0) we observe a field intensity peak of 1.08 MV/m, as shown in Figure 2h. This field intensity is more than 2 times lower than that for the internal cap‐gap field and nearly 5.2 times lower than for the peripheral cap‐gap field. Also, the transmittance at resonance, given in Figure 2i, is ‐15.5 dB, which is more than 2.4 times (in dB) lower than for the octa‐structure MM.

We also calculated and compared the Q‐factor of the octa‐structure MM with the other MM architectures. The octa‐structure MM has a 3 dB bandwidth of 23 GHz at the resonating frequency of 700 GHz calculated from Figure 2c, with a Q‐factor of 30.43. In comparison, the arrowhead MM has a Q‐factor of 15.90 (3 dB bandwidth of 50 GHz) at the resonating frequency of 795 GHz calculated from Figure 2f, and the linear SRR has a Q‐factor of 7.88 (3 dB bandwidth of 85 GHz) at the resonating frequency of 670 GHz calculated from Figure 2i. This simulated Q‐factor of 7.88 for the linear SRR exactly matches the Q‐factor given in the literature.^[^ ^35^, ^55^ ^]^

These comparisons with the well‐known MM architectures for dielectric sensing indicate that our octa‐structure MM has a considerably higher electric field at the cap‐gaps and a high Q resonance. This is possible due to the design of the MM, accompanied by much larger overall cap‐gap areas, and, more importantly, efficient utilization of the hotspots as peripheral cap‐gaps. By not placing the octahedral unit cells in a clustering fashion, we can achieve a transmittance of only ‐28 dB at resonance, indicating much lower plasmonic interaction on the metasurface (electric field and transmission parameter simulation results for an octa‐structure MM not arranged in a honeycomb fashion are given in supplementary information section 2). Since the resonator is purely ELC with net zero magnetic coupling, the plasmonic interaction occurring in the cap‐gap regions contributes to the red‐shift of the resonance frequency, and negligible energy is absorbed by the MM.^[^ ^53^, ^54^ ^]^ Therefore, we can expect much higher dielectric sensitivity with the octa‐structure MM compared with the other architectures.

**Figure 3:**
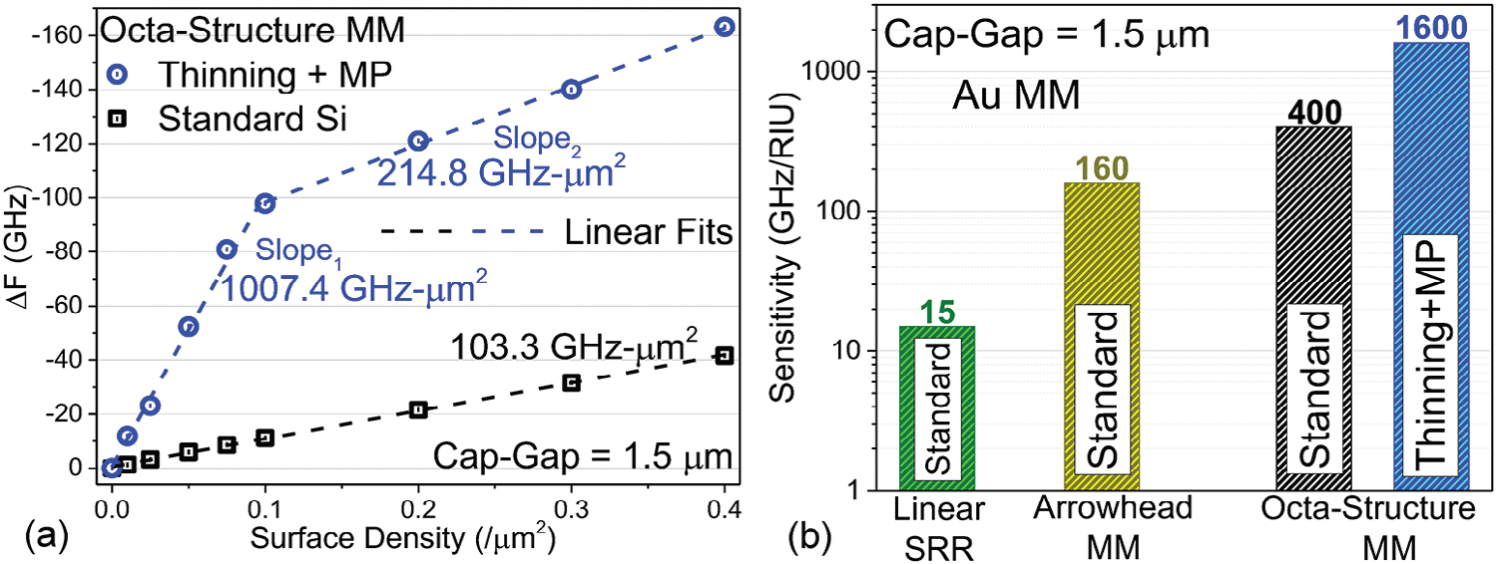
(a) Simulated Δ*F* is plotted against surface density of the dielectric placed on the metasurface for both standard Si substrate, 525 µm thick Si (black line) and 200 µm thinned Si with BP attached (blue line). The slopes of both curves are also labelled. (b) Calculated sensitivity values are plotted and compared in bar graph for linear SRR with standard Si (green bar), arrowhead MM with standard Si (yellow bar), and octa‐structure MM with standard Si (black) and thinning + BP configuration (blue).

To predict the sensitivity and the increased dynamic range response due to the dielectric material spread on the metasurface, we simulated Δ*F* for low to moderate surface densities of dielectric. The dielectric units in the simulation were composed of 100 nm–length cubes (nanoparticles), spread throughout the active area^[^ ^32^, ^33^, ^34^ ^]^ (for details of the simulations with dielectrics, see supplementary information section 3). The surface density was varied by varying the fill‐factor (FF) of the nanoparticles between ultra‐low density (0.01 particles/µm^2^ to 0.1 particles/µm^2^), low density (0.1 particles/µm^2^ to 0.2 particles/µm^2^) and moderate density (0.3 particles/µm^2^ to 0.4 particles/µm^2^), as shown in **Figure** **3**A. Thereby, Δ*F* is calculated by subtracting the resonance frequency of the MM with the dielectric cubes from the resonance frequency of the pristine sample (without dielectric). A similar method of categorizing dielectric surface density was used in our previous work as well.^[^ ^33^ ^]^ This method of studying the dielectric induced Δ*F* enables precise comparison of the simulated Δ*F* with the spectroscopic Δ*F*, by correlating the simulated surface density of the particles (dielectric) with the dielectric concentration of proteins/nanoparticles used in the experiments.

Figure 3a shows the simulated Δ*F* for various surface densities of dielectric cubes on an octa‐structure MM with a cap‐gap of 1.5 µm, fabricated on a standard Si substrate of typical thickness 525 µm (for 4‐inch wafers). The results are represented by black hollow squares, linearly fitted with a dashed line. At an ultra‐low surface density of 0.01/µm^2^, we observe a Δ*F* of ‐1.32 GHz, which increases to ‐41.6 GHz for a FF of 0.4/µm^2^. The Δ*F* versus surface density plot for a 52 5µm thick Si substrate has a perfectly linear trend, as expected, before reaching saturation (Δ*F_sat_* for high concentrations of dielectric,^[^ ^35^, ^37^ ^]^) with the slope of the linear fit given by *slope*_525_ =  103.3 *GHz* − µ*m* ^2^.

In our previous work, we reported on two separate ways of FP‐MM resonance decoupling from high permittivity substrates, which results in MM surface plasmonic enhancement and consequent multi‐fold dielectric sensitivity enhancement.^[^ ^34^ ^]^ One of the methods involves thinning the Si substrate down to 200µm, which broadens the FP periodicity, thereby reducing the probability of FP‐MM resonance coupling.^[^ ^34^ ^]^ The other method involves optically attaching dielectric back‐plates (BPs) or Meta‐Plates (MPs) of specific thickness to the backside of the MM chip, which considerably reduces the absorption in the FSR regions, thereby minimizing the substrate losses.^[^ ^50^ ^]^ A combination of thinning and BP increased the dielectric sensitivity by 8.2 times for an arrowhead MM.^[^ ^50^ ^]^ We have applied the above sensitivity enhancement techniques on our octa‐structure MM, where we thinned the 525 µm substrate to 200 µm, and attached the dielectric BP optically to the backside (350 µm of quartz and 500 µm of PTFE), to simulate the total possible Δ*F* enhancement. We plotted the simulated Δ*F* for various surface densities with the thinning and BP combination, represented by blue hollow circles linearly fitted with a blue dashed line, in Figure 3a. For an ultra‐low FF of 0.01/µm^2^, we observe a Δ*F* of ‐11.9 GHz, which is more than 10 times greater than in the standard Si substrate case; Δ*F* increases up to ‐163.2 GHz for a FF of 0.4/µm^2^, which is nearly 4 times higher than for the standard Si substrate thickness for a moderate FF. The thinning and BP attachment increases the Δ*F* for an ultra‐low concentration FF more drastically than for a low to moderate FF.^[^ ^33^ ^]^ Due to this, we can observe a change in the slope.^[^ ^33^ ^]^ Between the surface densities of 0.01/µm^2^ and 0.1/µm^2^ (extremely low dielectric concentration) a perfect linear fit can be drawn with *slope*_*Thinning* + *MP*_1 = 1007.4 *GHz* − µ*m* ^2^, and between 0.1/µm^2^ and 0.4/µm^2^ (low to moderate dielectric concentration) another perfect linear fit can be drawn with a lower slope value – *slope*_*Thinning* + *MP*_2 = 214.8 *GHz* − µ*m* ^2^.

Linear fitting and slope calculation enables the calculation of dielectric sensitivity values. By fitting the data with the relation $\Delta F\;=\;\Delta F_{sat}\left(1-e^{-\frac{Particle\;Concentration}{Particle\;Concentration_{sat}}}\right)$, the sensitivity can be estimated in terms of particle concentration, where Δ*F_sat_* is the saturated Δ*F* with the maximum (saturated) particle concentration.^[^ ^33^, ^35^ ^]^ However, sensitivity is calculated here in terms of the refractive index unit (RIU) since this is the common unit used to calculate and compare sensitivity. Effective RI after adding the external dielectric (*n*_*eff* − *dielectric*_) is calculated using the formula, $n_{eff-dielectric}=\sqrt{\varepsilon_{eff}+\alpha N\left(\varepsilon_{f}-1\right)}$, where ε_*eff*_ is the effective dielectric constant without the deposition of the dielectric, α is the coefficient which is associated with the surface fraction of the nano cubes, ε_*f*_ is the dielectric constant of one block of the cube, and *N* denotes the number of dielectric particles in the active area;^[^ ^37^ ^]^ from the *n*_*eff* − *dielectric*_ value, the sensitivity ratio of Δ*F* to RIU is obtained. In Figure 3b we present the calculated sensitivity of the octa‐structure MM (with standard Si substrate and thinning + BP configuration) together with results for the arrowhead MM and linear SRR with identical cap‐gaps of 1.5 µm in all MMs (100 nm of Au metal and 525 µm of Si substrate) for an FF range between 0.1/µm^2^ and 0.4/µm^2^. Complying with the previously published results, the linear SRR has a dielectric sensitivity of 15 GHz/RIU,^[^ ^35^ ^]^ while the arrowhead MM has a sensitivity of 160 GHz/RIU.^[^ ^32^ ^]^ The octa‐structure MM with a standard Si substrate of 525 µm thickness has a dielectric sensitivity of 400 GHz/RIU, which is 2.5 times higher than for the arrowhead MM, and more than 26 times higher than for the classical linear ELC SRR. Upon thinning the Si substrate to 200 µm and optically attaching the BP, we observe a huge increase in dielectric sensitivity to 1600 GHz/RIU.

These simulations demonstrate that our new octa‐structure MM is potentially ultra‐sensitive to any dielectric, ranging from a very low to a moderate FF, owing to its larger effective cap‐gap areas and efficient utilization of the peripheral hotspot as a cap‐gap for dielectric sensing. In the next section we compare our simulated results with THz impedance spectroscopic analysis done in the laboratory.

## 4 Experimental Results and Discussions

**Figure 4:**
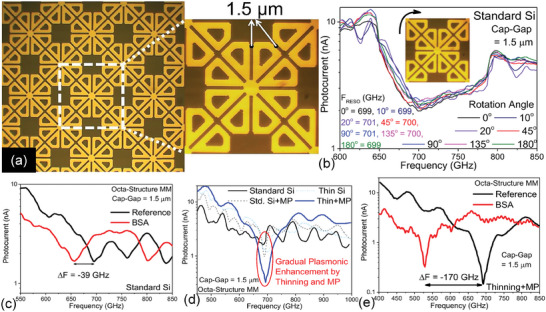
(a) Microscopic image of the octa‐structure MM with magnified inset showing clear demonstration of the octa‐structure unit cells arranged in clustered fashion with peripheral hotspots transformed into cap‐gaps of 1.5 µm. (b) Experimental transmission parameters (in photocurrents) plotted for different rotation angles of the MM with standard Si substrate (525 µm thick). F_RESO_ at all rotation angles are marked. (c) THz transmission spectra for octa‐structure MM on standard Si substrate with 0.001% BSA, showing Δ*F* of ‐39 GHz. (d) THz transmission spectra for octa‐structure MM showing gradual plasmonic enhancement (enhanced resonance dip) for standard substrate by thinning the Si to 200 µm and by attaching the MP (BP). (e) THz transmission spectra for octa‐structure MM with thinning + MP (BP) configuration with 0.001% BSA, showing increased Δ*F* of −170 GHz.

The octa‐structure MM with a 1.5 µm cap‐gap used for the impedance spectroscopy measurements was fabricated on 4‐inch Si wafers with a standard thickness of ∼525 ± 25 µm and diced into 8 × 8 mm chips (periodic metasurface area, 6 × 6mm), with each chip containing more than 9,500 nano‐antenna structures, (see supplementary information section 4 for details of the fabrication procedures). Further, for experimental purposes, we performed mechanical polishing to thin down the Si substrate to 200 µm and attached the BP (see supplementary information Sections 5 and 6 for details regarding the thinning and BP attachment process). **Figure** **4**a shows a microscopic image of the fabricated octa‐structure MM with a zoom‐in showing how the unit cells are closely connected with one another homogenously, to effectively utilize the peripheral field hotspots for dielectric sensing.

We performed spectral scanning of the octa‐structure MM, exploring the changes in the resonance frequency with the incident light polarization. We used a linearly polarized THz spectrometer in transmission mode to illuminate the metasurface from the front (see supplementary information section 7 for details of the THz spectrometer), and rotated the MM chip from 0° to 180°, with 45° intervals, and also included arbitrary rotation angles of 10° and 20°. Physical rotation of the metasurface when exposed to linearly polarized light helps to prove the polarization independence of our metastructure. THz transmittance spectra are plotted in terms of photocurrent, in Figure 4b, and are recorded from the spectrometer's receiver without any further processing. We can observe in Figure 4b that the transmittance graphs nearly overlap with one another when the metastructure is rotated from 0° to 180°, with 45° intervals and the 10° and 20° arbitrary angles. The *F_reso_* slightly varies from 699 GHz to 701 GHz with the rotation, so the mean F_RESO_ remains at 700 GHz with an error of ±1 GHz. Hence, we can infer that the octa‐structure resonator is a super‐symmetric structure that is independent of the incident light's polarization.

To experimentally validate the resonance spectral shift values calculated in the simulations, we performed THz impedance transmission spectroscopy of the octa‐structure MM devices, deploying Bovine Serum Albumin (BSA) protein solution as the dielectric for the cap‐gaps (see supplementary information section 8 for details regarding the BSA solution preparation). In all the spectral data, the spectra of the pristine samples are shown in black, and their respective measurement spectra after depositing the dielectric are shown in red. Δ*F* is calculated by subtracting the measurement resonance frequency from the reference resonance frequency dips, giving a negative value for all red shifts. Figure 4c shows a spectral Δ*F* of ‐39 GHz with 0.001% dry BSA solution for an octa‐structure MM fabricated on a standard Si substrate (525µm thickness). This experimental Δ*F* is well correlated with the simulated Δ*F* of ‐41.6 GHz for a moderate FF of 0.4/µm^2^ (Figure 3a). Compared to other resonator architectures published in the literature, We obtained a Δ*F* increase from 12 GHz with 0.001% BSA solution^[^ ^34^ ^]^ in the arrowhead architecture to ‐41.6 GHz, for the octa‐structure MM. This improvement in dielectric response is due to increased cap‐gap areas and the increased field intensity, specifically in the peripheral cap‐gap hotspots.

We now apply our previously published sensitivity enhancement techniques of thinning and optical attachment of BP on the octa‐structure MM to maximize Δ*F* ^[^ ^34^, ^50^ ^]^ and the Q‐factor. In Figure 4d, we show the plasmonic enhancement effect of the electric field on the metasurface after thinning the substrate to 200 µm and attaching the BP. Compared to the standard Si substrate of 525 µm thickness (solid black line), the addition of the BP only deepens the resonance (grey dashed line) and improves the Q‐factor. Thinning of the substrate to 200 µm without addition of the BP (light blue dotted line) also deepens the resonance compared to the standard Si of 525 µm thickness. Finally, a combination of thinning and BP (solid blue line) drastically deepens the resonance and considerably improves the Q‐factor. The Q‐factor is calculated by obtaining the 3 dB bandwidth from the SNR of the experimental spectra (the noise value of the receiver of the spectrometer is given in supplementary information section 7). The MM on the standard Si substrate of 525 µm has a Q‐factor of 29.41 (3 dB bandwidth is 23.8 GHz at 700 GHz, calculated from Figure 4c,d), which is close to the simulated Q‐factor of 30.43 from Figure 2c. The combination of thinning and BP vastly improves the Q‐factor to 120.69 (3 dB bandwidth is 5.8 GHz at 700 GHz, calculated from Figure 4e).

The spectrometric Δ*F* for the thinning and BP combination, with the same 0.001% BSA solution deposited on the octa‐structure metasurface, is improved to −170 GHz (Figure 4e), which is well correlated with the simulated Δ*F* of −163.2 GHz for 0.4/µm^2^ FF. This is exactly a 4.25 times improvement in resonance spectral shift compared to the standard Si substrate. Hence, we again prove the claim made in our previous works that these plasmonic enhancement techniques of thinning and BP attachment can be applied on any resonant MM to remove the substrate losses and maximize their dielectric response.^[^ ^34^, ^50^ ^]^ Therefore, in this work, we also propose to use the sensitivity enhancement techniques in conjunction with our newly developed octa‐structure MM to create a composite structure for ultrasensitive particle sensing (dielectric response).

**Figure 5:**
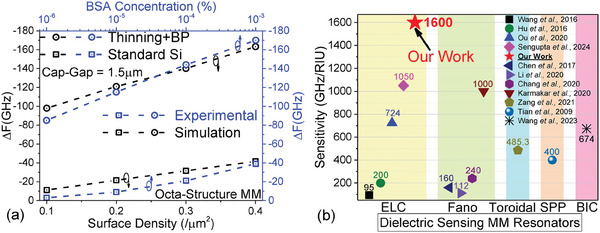
(a) Simulated Δ*F* (black data points) with varying surface density (FF 0.1/µm^2^ to 0.4/µm^2^) is plotted on the same graph with experimental Δ*F* (blue data points) with BSA concentration densities 10^−3^% to 10^−6^% for both standard Si substrate and thinning + BP configuration, for easy comparison between simulations and experiments. (b) Dielectric sensitivity of our octa‐structure MM with maximized sensitivity is compared with different types of dielectric sensing MM resonators. All the referenced works have a sensitivity magnitude label, where our work, denoted with a red colored star, has a sensitivity of 1600 GHz/RIU.

To quantify the Δ*F* enhancement due to using the octa‐structure MM, we performed a series of impedance spectroscopic measurements with varying BSA concentrations, from diluting to 10^−3^% up to 10^−6^%. Each Δ*F* data point was repeated five times, and its statistical average is plotted in **Figure** **5**a. We also plotted the simulated Δ*F* (as shown in Figure 3a) with different axes on the same graph to enable easy comparison between the simulation and the actual impedance spectroscopic experimental results. From the data in Figure 5a we can calibrate the experimental Δ*F* values in a way that 10^−3^% BSA correlates with the simulated Δ*F* values of 0.4/µm^2^ FF. Similarly, experimental Δ*F* for 10^−4^% BSA correlates with the simulated Δ*F* for 0.3/µm^2^ FF, Δ*F* for 10^−5^% BSA correlates with the Δ*F* for 0.2/µm^2^ FF, and Δ*F* for 10^−6^% BSA correlates with the Δ*F* for 0.1/µm^2^ FF. For standard Si substrate (525 µm thickness), we observe a corresponding linear trend between simulations and experiments for 10^−3^% to 10^−4^% BSA concentrations (moderate FF 0.4/µm^2^ to 0.3/µm^2^), and slightly reduced experimental results for Δ*F* compared with simulations for 10^−5^% to 10^−6^% BSA concentrations (low FF 0.2/µm^2^ to 0.1/µm^2^). In fact, we can observe 10 GHz lower Δ*F* in experiments specifically for standard Si substrate (525 µm thickness). This difference occurs due to frequency bifurcation and anchoring effects for thicker substrates.^[^ ^34^ ^]^ Also, for standard Si, a small change in the die thickness (if the die is taken from different parts of the wafer) causes the resonance frequency to differ by ±25 GHz.^[^ ^34^ ^]^ Due to this phenomenon, calibration of experimental Δ*F* values compared to the simulation becomes extremely difficult, specifically at lower dielectric concentrations.

Upon enhancing the sensitivity by thinning the Si substrate to 200µm and BP attachment on the backside, we can observe much better agreement between experiments and simulations, due to reduced substrate losses. We again observe a similar linear trend for Δ*F* between simulations and experiments for 10^−3^% to 10^−4^% BSA concentrations (moderate FF 0.4/µm^2^ to 0.3/µm^2^). For concentrations of 10^−5^% to 10^−6^% BSA (low FF 0.2/µm^2^ to 0.1/µm^2^), the experimental Δ*F* is slightly lower than the simulated ones: <10 GHz lower than simulated values for 10^−5^% BSA concentration and 6 GHz lower than simulated values for 10^−4^% BSA concentration. Despite these minor discrepancies between simulated and experimental data, both the values conform to the linearly increasing nature of Δ*F* when plotted against increasing dielectric concentration.

This correlation between simulated and experimental Δ*F* data with respect to varying dielectric concentration offers an opportunity to calculate and predict dielectric sensitivity by simply performing simulations with different FF values. Once the experimental dielectric concentrations have been calibrated against the equivalent FF value in the simulations, one can extrapolate and predict the Δ*F* for extremely low concentration dielectrics; this is especially pertinent for medical applications like breathalyzer‐based sensing of lung diseases.^[^ ^32^, ^33^ ^]^ Thanks to this calibration, we claim that the sensitivity values presented in Figure 3b will reasonably correspond to the experimental sensitivity values.

Since this simulated dielectric sensitivity correlates with our statistical experimental results extremely well, it is fair to conjecture that the simulated sensitivity will be the same as the experimental sensitivity. In Figure 5b we compare the sensitivity of our proposed setup with that of different types of MM resonators used in the dielectric sensing. In the first category, we show five examples of ELC resonators, including our work. Wang et al., showed a polarization sensitive ELC SRR for BSA detection with a sensitivity of 95 GHz/RIU.^[^ ^56^ ^]^ A slightly higher sensitivity, of 200 GHz/RIU, was demonstrated by Hu et al. with an absorber configuration with a microfluidic channel.^[^ ^57^ ^]^ Significantly higher sensitivity, of 724 GHz/RIU, was demonstrated by Ou et al. with a multi‐band THz resonance MM in transmission mode.^[^ ^58^ ^]^ In this work, we have used the sensitivity enhancement technique applied to the arrowhead MM (of thinning the Si substrate) to detect and screen multiple respiratory diseases, and achieved a sensitivity of 1050 GHz/RIU.^[^ ^33^ ^]^ In the second category of resonators, we examined the Fano resonator, which is an asymmetric MM exhibiting a special dark‐mode. These resonators generally feature high dielectric sensitivity: Chen et al., demonstrated a sensitivity of 160 GHz/RIU with a symmetric double cut wire;^[^ ^59^ ^]^ Li et al., demonstrated a sensitivity of 112 GHz/RIU with a reflective Fano resonator for ethanol detection;^[^ ^60^ ^]^ Cheng et al., demonstrated a sensitivity of 240 GHz/RIU with an ultrathin substrate;^[^ ^61^ ^]^ and Karmakar et al., demonstrated the highest sensitivity for Fano resonance of 1000 GHz/RIU with a stacked nano‐rod configuration.^[^ ^62^ ^]^ Among other types of resonators examined, we note a toroidal resonator with a quartz substrate that had a sensitivity of 485.3 GHz/RIU,^[^ ^28^ ^]^ surface plasmon polariton (SPP) sensors with a metal‐hole structure that had a sensitivity of 400 GHz/RIU,^[^ ^63^ ^]^ and quasi bound states in continuum (quasi‐BIC) sensors with a magnetic dipole that had a relatively higher sensitivity of 674 GHz/RIU.^[^ ^64^ ^]^ In this work we have deliberately used BSA protein to calculate the sensitivity parameters of our octa‐structure MM so that we can have a fair comparison of all the sensitivities of the recent works we cited here. Most of the recent works used some form of organic substances like proteins or cells, or sugar solution, which also has a very similar dielectric constant to organic substances (30 to 50).^[^ ^65^ ^]^ This allows us to make a proper comparison in Figure 5b.

The proposed octa‐structure MM ELC sensor achieved a high sensitivity of 1600 GHz/RIU (enhanced by thinning + BP), which is higher than all existing types of MM resonators suitable for dielectric sensing. We have studied the latest works – mostly published between 2016 and 2024 – including solutions based on ultra‐thin substrates, asymmetric MM structures, toroidal structures, and quasi‐BIC resonators, which are relatively difficult and costly to fabricate compared to simple ELC resonators. Moreover, our resonator is super‐symmetric and can be used with any polarization type of THz sources. More importantly, our ELC resonator is CMOS compatible, only involving standard fabrication processes of photolithography, lift‐off, polishing, and optical connection of external substrates. Therefore, this new proposed octa‐structure MM will be suitable for any application that requires ultra‐sensitive dielectric sensing, including the sensing of nanoparticles and biomolecules, and the detection of viruses, bacteria, and other biological samples.

## 5 Conclusion

In this work, we have developed a new highly sensitive ELC THz resonator with an octa‐structure architecture. This new super‐symmetric architecture has a much larger active area for dielectric sensing (cap‐gap areas) compared to other ELC resonators. It also utilizes the peripheral high electric field regions by placing the unit cells strategically close to each other so that the spacing between the octa‐structure becomes an active area with the same cap‐gap. The peripheral cap‐gap has 2.5 times more field intensity compared to the internal cap‐gap, which makes these interconnecting capacitors true hotspots for dielectric sensing. The architecture has much lower inductive elements, achieved by deliberately removing the metals from lobes of the octa‐structure MM surface. Simulations, backed by impedance spectroscopic analysis and experimental results, reveal that the dielectric sensitivity of our proposed octa‐structure MM is 400GHz/RIU, which is 2.5 times higher than for the arrowhead MM, and more than 26 times higher than for the well‐known linear ELC SRR. Further sensitivity enhancement using our previously established techniques of thinning and BPs increases the sensitivity of the same octa‐structure MM to 1600 GHz/RIU, which is one of the highest recorded sensitivities for a dielectric sensing MM resonator to date with a high‐Q resonance of 120.69. Upon comparing our work with the latest works of different genres of THz dielectric sensor, mostly published within the years 2016 to 2024, we can claim that we have developed an excellent MM for highly sensitive particle sensing. Our sensor is fully CMOS compatible and will be suitable for any application that requires ultra‐sensitive dielectric sensing, including the characterization of nanoparticles and biomolecules, and the detection of viruses, bacteria and any other biological samples.

## Conflict of Interest

The authors declare no conflict of interest.

## Supporting information



Supporting Information

## Data Availability

The data that support the findings of this study are available in the supplementary material of this article.
