# Supplementary material for: Novel Octa‐Structure Metamaterial Architecture for High Q‐Factor and High Sensitivity in THz Impedance Spectroscopy
Source: Adv Sci (Weinh). 2024 Oct 30;11(47):2407824. doi: 10.1002/advs.202407824 (PMC11653601; doi:10.1002/advs.202407824)
Supplement: Supplementary file 1 — Supporting Information [file ADVS-11-2407824-s001.pdf]

ADVANCED SCIENCE
Open Access

Supporting Information

for *Adv. Sci.*, DOI 10.1002/advs.202407824

Novel Octa-Structure Metamaterial Architecture for High Q-Factor and High Sensitivity in THz Impedance Spectroscopy

*Heena Khand, Rudrarup Sengupta and Gabby Sarusi**

Novel Octa-Structure Metamaterial Architecture for High Q-Factor and High Sensitivity in THz Impedance Spectroscopy

Supporting Information

Heena Khand, Rudrarup Sengupta, and Gabby Sarusi*

Department of Photonics and Electro-Optics Engineering, School of Electrical and Computer Engineering, Ben-Gurion University of the Negev, Beer Sheva, Israel.

*Correspondence author, Orcid ID: 0000-0001-6717-2235; email: sarusiga@bgu.ac.il

1: Architectural engineering and development of octahedral MM

The absorber eight sector structure for microwave frequencies had a circular shaped unit cell[1]. To make the unit cell easier for photolithography, we changed the shape to octahedron with equal sides. The unit cell diameter was chosen to be 60µm, with cap-gap of 1.5µm suited for THz frequencies with resonance frequency of 700GHz. To utilize the peripheral capacitive hotspots the unit cells are arranged in clustered fashion, as shown in figure S1(a). The simulated normalized S parameters are plotted in figure S1(b), showing a transmission (red line) dip near the resonance frequency a nearly zero reflectance (black line) and a nearly perfect absorbance (blue line) at the resonance region, as expected. The relative permittivity of the absorber eight-sector MM shown in figure S1(c) maintains a negative value for a large spectrum of THz frequencies including the resonance region. Furthermore, the imaginary permittivity remains quite high throughout the THz spectrum, which is an expected behavior from an absorber architecture. Although the capacitor gaps are quite enough to provide an increased capacitance, however the inductance is largely increased as well, in this structure. Moreover, lots of metals in the metasurface and uncancelled magnetic fields is responsible for huge THz radiation absorption in all THz spectra.

Contrastingly, when we remove the metals from the center of each lobe, we get our octahedral MM shown in figure S1(d), and we obtain a perfect ELC resonant architecture. The simulated normalized S parameters are plotted in figure S1(e), showing a transmission (red line) dip near the resonance frequency a nearly 100% reflectance (black line) and a nearly zero absorbance (blue line) at the resonance region, as expected. The relative electric permittivity plotted on figure S1(f) shows a typical and perfect behavior of an ELC resonator, where the permittivity decreases sharply at resonance region, and becomes negative just at the resonance region.

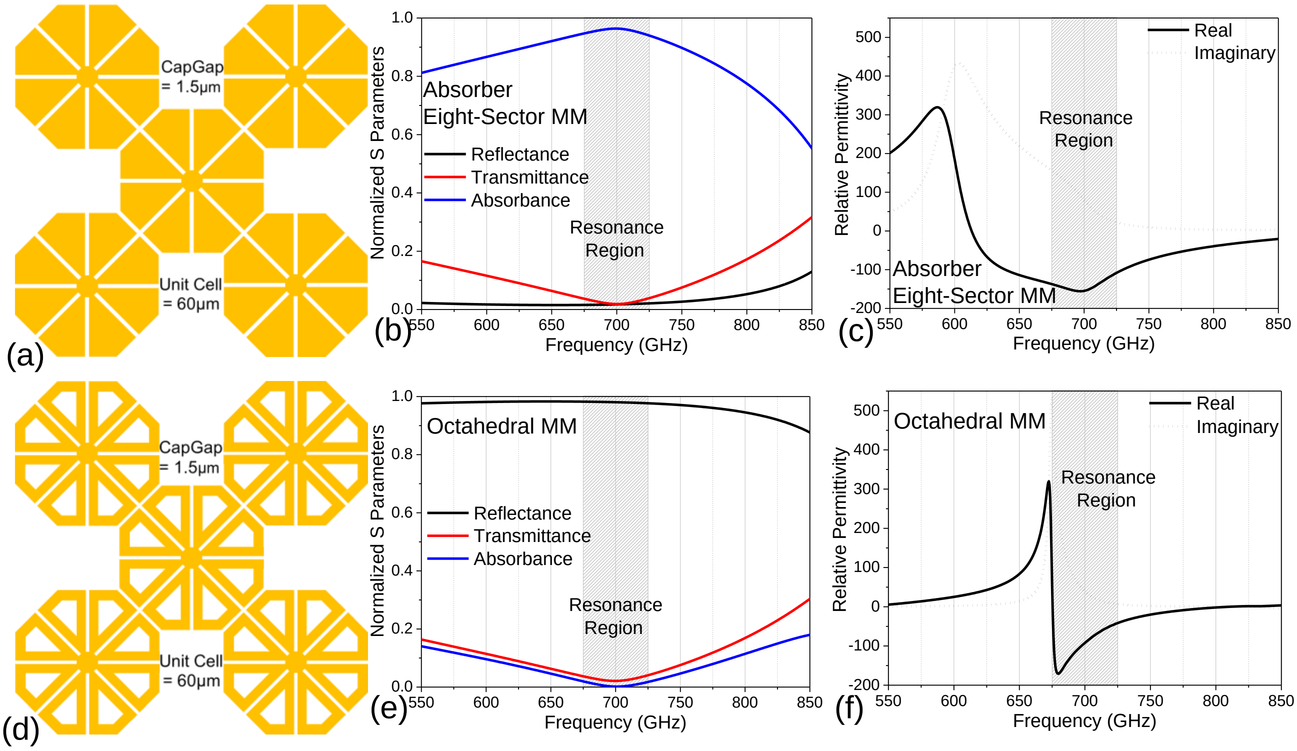

Figure S1. (a) Schematic structure of the absorber eight-sector MM. (b) Normalized S parameters are plotted, showing reflectance in black line, transmission in red line and absorbance in blue line. (c) Relative permittivity is poltted with real part shown in solid black line and imaginary part plotted in dotted black line. (d) Schematic structure of the octa-structure MM. (e) Normalized S parameters are plotted, with same nomenclature as in (b). (e) Relative permittivity is poltted with same nomenclature as in (c).

2: Arrangement of clustered octa-structure unit cells

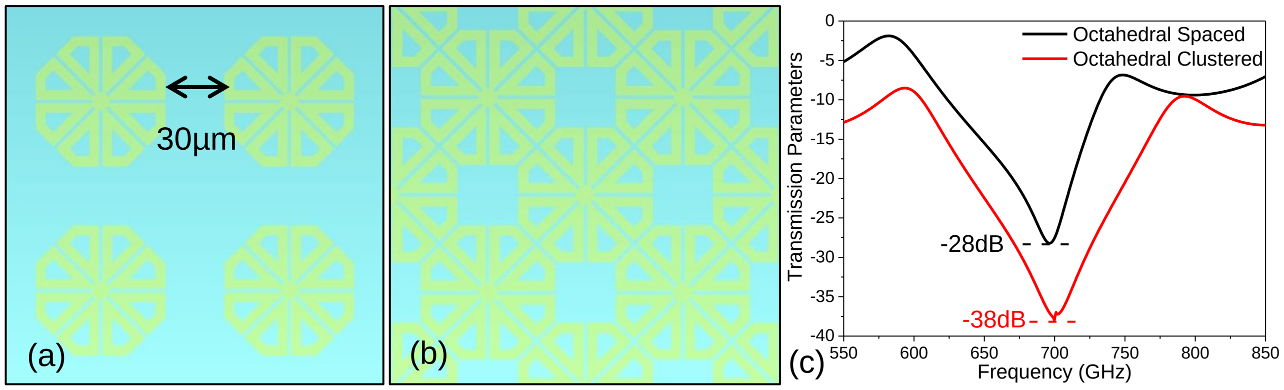

Figure S2. Schematic structure of the octahedral MM with (a) 30µm gap between each unit cells, and (b) aranged in clustered fashion utilising peripheral hotspots. (c) Transmission paameters of the octahedral spaced MM shown in black and octahedral clustered MM shown in red.

To investigate the effect of spacing between the octahedral unit cells, we have simulated two different cases. Figure S2(a) shows the first arrangement where the unit cells are placed at 30µm gap between each other, homogeneously. Figure S2(b) shows the octahedral unit cells arranged in

clustered fashion to utilize the peripheral hotspots for particle detection. The transmission parameters shown in figure S2(c) shows that the resonance transmission depth reached to only -28dB when the octahedral unit cells are spaced by 30µm gap, whereas when the octahedral unit cells are closely packed in clustered fashion, we get a resonance transmission depth of -38dB. Therefore, we can expect much larger dielectric sensitivity with the octa-structure MM packed densely in clustered fashion compared to what has been published in recent times.

3: Details regarding the Simulation with Dielectrics

The 3D simulation was done extensively using CST studio suite for analyzing the octa-structure metamaterial. First, we build and analyze the octa-structure MM (figure S3 (a)) which consists of a plurality of antenna structures.

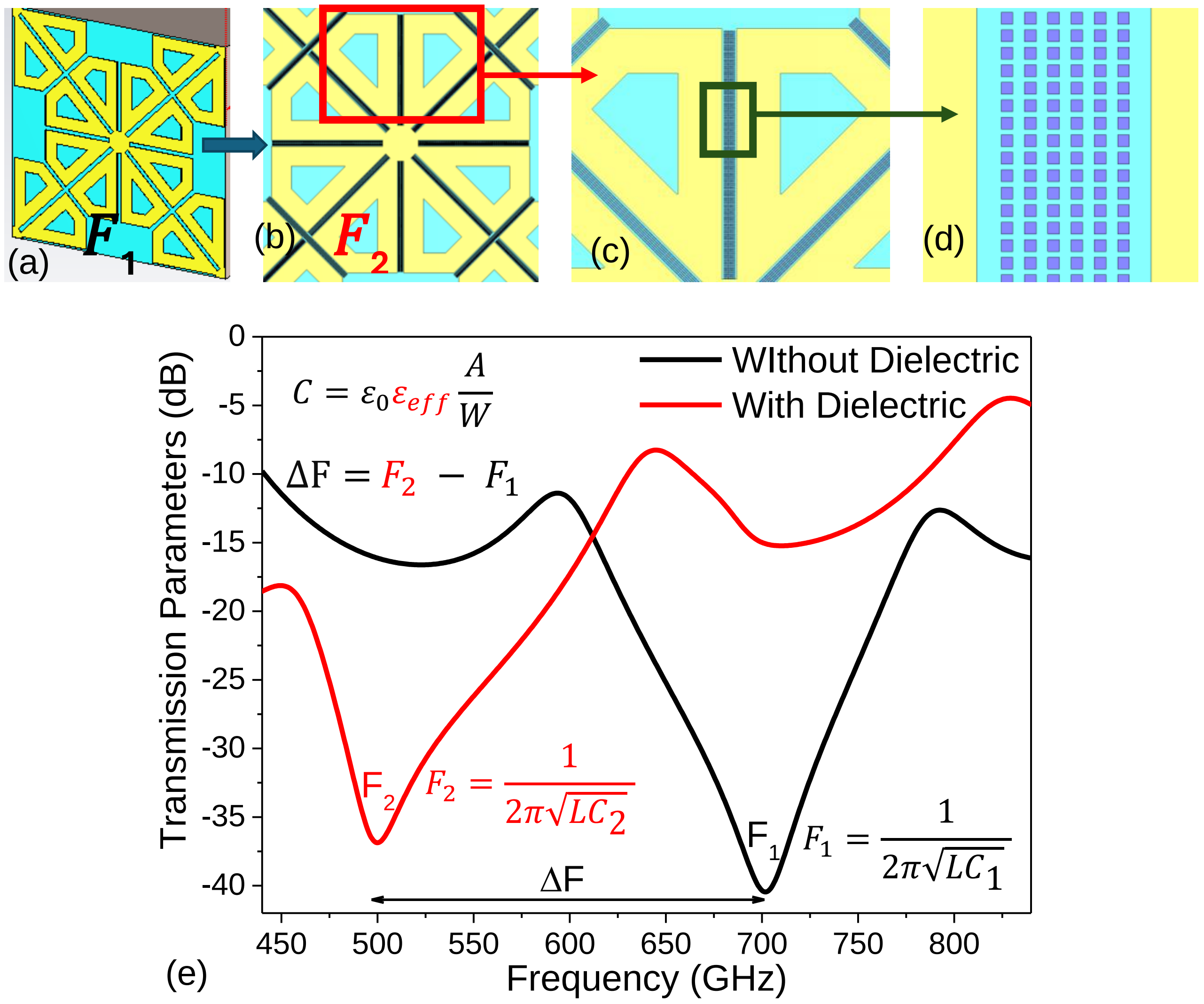

Figure S3. (a) The structure of octa-structure MM with 1.5µm cap-gap without any dielectric particle. (b) Illustrates the MM structure with cubic dielectric particles sized 100nm in length and (c) and (d) shows zoomed pictures

depicting how the cubes are placed in the cap-gap. (e) The transmission spectra of MM resonance for (a) and (b) cases show red-shift in frequency (-201 GHz) for dielectrics placed in cap-gap by the formulae shown.

To know the dielectric response of the MM, we design nanoparticle cubes (as analytes) of length 100nm and specific dielectric constant to distribute them within the capacitor-gap (active area) of the MM with a specific fill factor (FF) as shown in figure S3(b-d). The resonating frequency of the MM structure (in vacuum) is shown in the S-parameters in the transmittance graph shown in figure S3(e). The distribution/concentration of the cubes (figure S3(c-d)) and their dielectric constant can be varied accordingly. The presence of analytes in the cap-gap (in comparison to the structure without analyte in figure S3 (a)) will result in a red shift of the resonance frequency, due to an increase in the capacitance of the capacitors; the shift in frequency ΔF (dielectric response) is shown by figure S3(e) and also the equations are added to understand the red-shift in frequency.

We use cubes with a dielectric constant of 40 for this work, without adding any loss factor. This is done to mimic proteins, in our case Bovine Serum Albumin (BSA). Most proteins have a very high dielectric constant in the range of 30 to 60 and at THz frequencies, the loss factor for such dielectrics is close to zero (negligible). Note that in this particular simulation, the dielectrics were deliberately put in high concentration/quantity to that the arrangement of nano-cubes in the active area are well understood.

4: Fabrication methods and working prototype

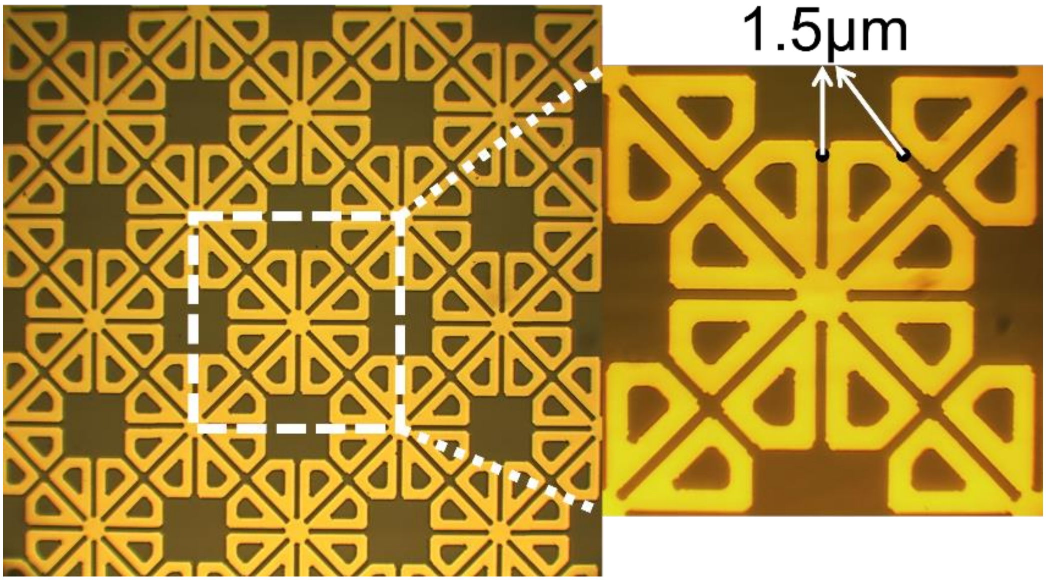

Figure S4. Microscopic image of the fabricated octahedral LC resonant MM structure on Si chip.

Octahedral MM structure with 1.5μm cap-gap width used for the impedance spectroscopy measurements was fabricated on a 4-inch Si wafer having a standard thickness of 525±25μm and

diced into 8mm squares chips, with each chip containing more than 9,500 nano-antenna structures forming the MM surface. The chips are prepared by e-beam evaporation and standard lithography. Figure S4 shows a microscopic image of our fabricated octa-structure LC resonant MM patterns. A 100nm Gold film was deposited by e-beam evaporation to define arrays of electrical arrowhead resonators with a line width of 4μm, outer dimensions of 60μm × 60μm, and a cap-gap of 1.5μm.

5: Polishing and thinning of substrate

We use the automatic and the manual polisher to thin down the conventional 525μm thick Si substrate to 200μm thickness. Note that we covered the antennas with a protective film to avoid any damage and polished the die from the backside. We put the chip on the stub as shown in figure S5(b) such that the substrate is on the top side as shown in the picture. We use 3 different types of polishing paper grades to thin down the Si substrate and then to mirror-polish the surface. While working with the automatic polisher, we keep the minimum pressure for a longer time to thin accurately, and consequently mirror-polish in the manual polisher.

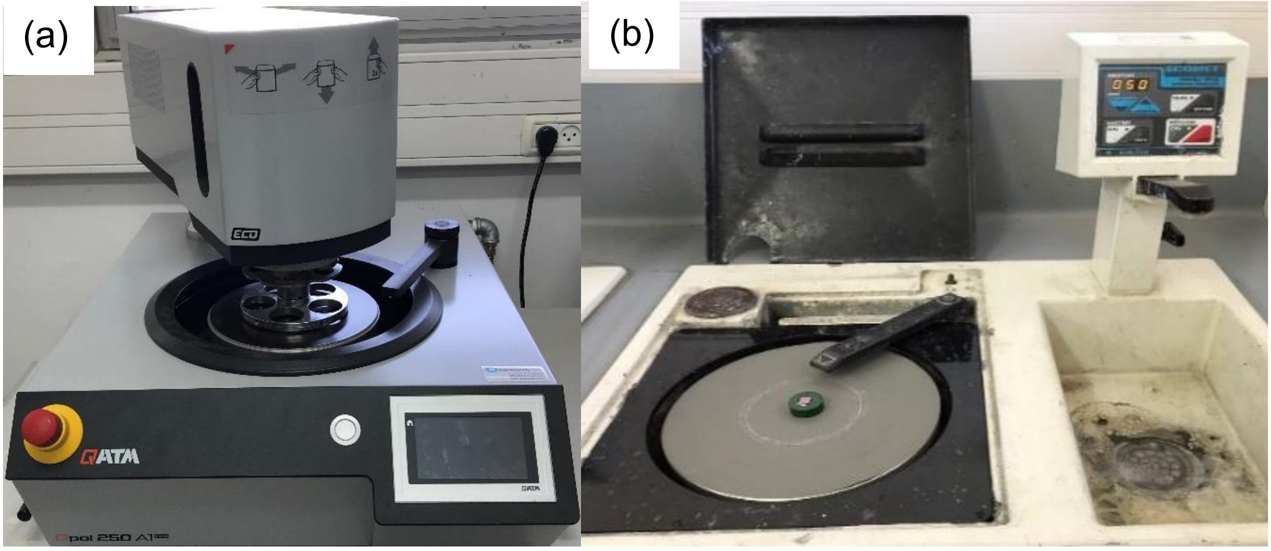

Figure S5. (a) Automatic polisher for thinning 725μm thick Si to 200 μm (b) manual polisher used for thinning and polishing the chip placed (MM structures facing down) on the green stub as shown.

6: Cascading of Substrates

To optically connect the back-substrate with the Si, we use optical glue, that cures on exposure to ultra-violet radiation. A thin layer of the glue is applied on the back-substrate and stuck onto the backside of the Si. A UV light source is used from the top to cure the glue. The same phenomeon is applied to optically connect the quartz and PTFE substrates as well.

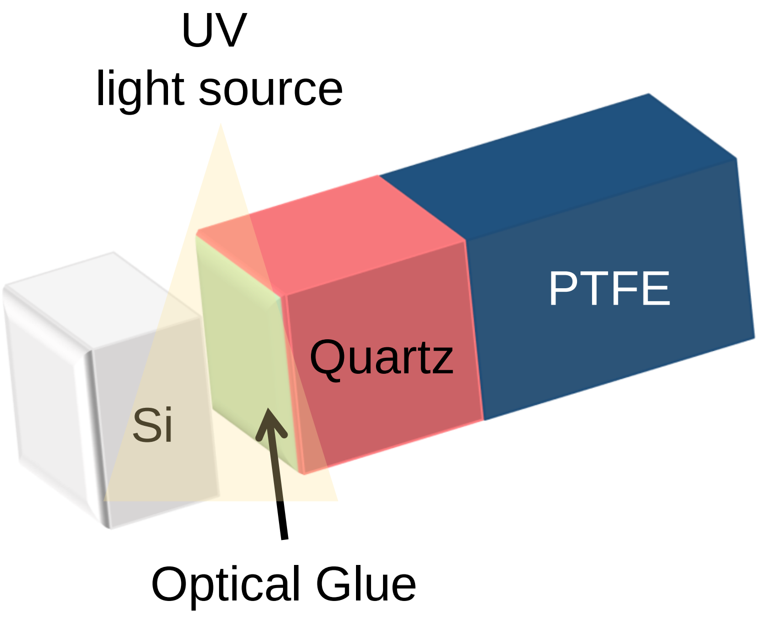

Figure S6. Experimental mechanism to optically connect the back-substrate (quartz and PTFE) to the backside of Si substrate

To prepare 350μm of quartz plate for 200μm thick Si, multiple thin quartz substrates from 4-inch quartz wafers (X-cut ad ST-cut) are optically connected with optical glue. The PTFE substrate of desired thickness is 3D printed and optically connected with the quartz plate. Finally, the entire back-substrate plate is cut to the exact size of the Si dies (8mm by 8mm) to be cascaded with the Si substrate (Figure S6).

7: Information about THz spectrometer used

We used a linearly polarized Toptica Systems TeraScan 1550 to record the transmittance spectra. This spectrometer has an InGaAs photomixer with a metal-insulator-metal heterostructure architecture. The photomixers use distributed laser feedback (DFB) technology to unite two temperature-controlled 1.5um lasers with a minute difference in wavelength and obtain the envelope of the interference spectrum, termed the 'laser beat', which is in the THz domain[2]. This spectrometer works with a coherent detection scheme, where the second photomixer acts as the THz receiver. The incoming THz wave generates a voltage in the antenna, while the 'laser beat' modulates the conductivity of the photomixer[2]. The resulting photocurrent (which is the unit of the output spectra) is proportional to the amplitude of the THz electric field[2]. The entire setup is controlled by a microcontroller unit (MCU) based on a FPGA with an internal clock rate of 130MHz to facilitate different programming operations[2]. Figure S7 shows our entire THz scanning setup for screening coronavirus carriers. The receiver has a noise of 12pA at 300ms lock-in integration time[2].

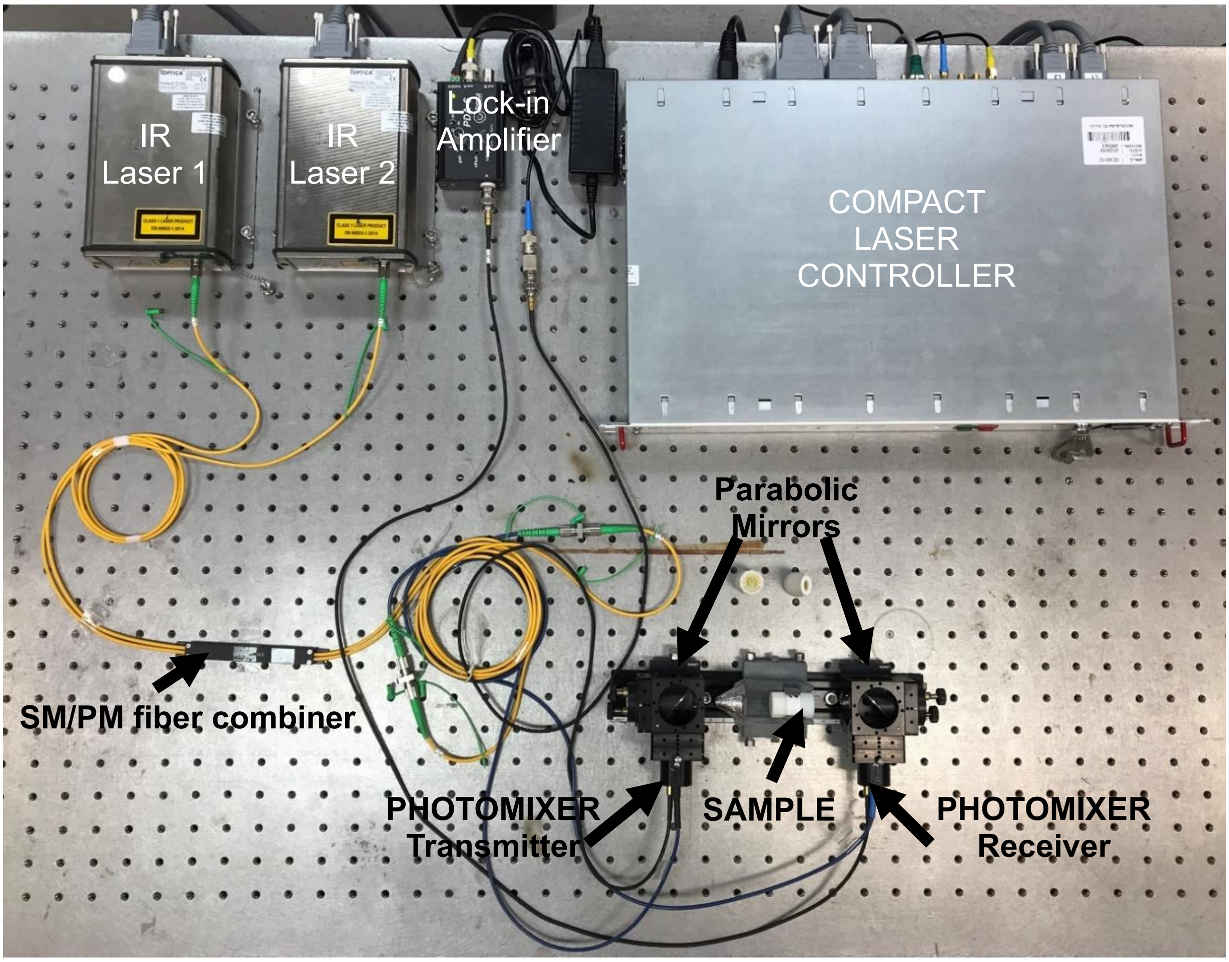

Figure S7. Snapshot of our entire THz scanning system used to screen coronavirus and related particles.

8: Preparation of analytes for experiments

For the experiments, we use protein nanoparticles of Bovine Serum Albumin (BSA), which needs to be dissolved and diluted in saline solution or de-ionized (DI), water in an organized way for our experiments. To dilute the 140gm of BSA particles, we use 1 liter of 0.9% saline solution. This solution is then diluted up to 0.001%. (0.9% saline means that in 100ml of DI water, we solubilize 0.9mg NaCl). We put 0.001% of BSA with the help of a pipette measuring 20μL on the MM chip surface and wait for the water to dry, analogous to drop and dry method. After these nanoparticles are dried on the surface, we check for the transmission spectra and subsequent dielectric response of the MM.

9: References

[1] T. T. Nguyen, S. Lim, *Sci. Rep.* **2017**, *7*, 3204.

[2] A. J. Deninger, A. Roggenbuck, S. Schindler, S. Preu, *J. Infrared, Millimeter, Terahertz Waves* **2015**, *36*, 269.
